# Supplementary material for: Classification of red beet and sugar beet for drought tolerance using morpho-physiological and stomatal traits
Source: PeerJ. 2025 Mar 21;13:e19133. doi: 10.7717/peerj.19133 (PMC11932113; doi:10.7717/peerj.19133)
Supplement: Supplemental Information 1 [file peerj-13-19133-s001.docx]

**Data codes:**

Column 1 shows “Replications” etc. 1, 2, 3, and 4.

Column 2 shows “Cultivars” whose names are inserted to the data file such as Bicores, BT Pancina, Yakut, Mohican, Orthega KWS, and Valentina.

Column 3 shows “Drought” with three levels: Control, 10% PEG and 20% PEG.

Please look at the column names for the investigated parameters.
